# Effectiveness of Ultrasound Therapy, TheraBite Device, Masticatory Muscle Exercises, and Stabilization Splint for the Treatment of Masticatory Myofascial Pain: A Randomized Controlled Trial

**DOI:** 10.1002/cre2.921

**Published:** 2024-06-24

**Authors:** Kenaz Salloum, Mawia Karkoutly, Ibrahim Haddad, Jihad Abou Nassar

**Affiliations:** ^1^ Department of Fixed Prosthodontics Damascus University Syria Syrian Arab Republic; ^2^ Department of Pediatric Dentistry Damascus University Syria Syrian Arab Republic; ^3^ Department of Biology Damascus University Syria Syrian Arab Republic

**Keywords:** myofascial pain, stabilization splint, TheraBite device, ultrasound therapy

## Abstract

**Background:**

Myofascial pain syndrome (MPS) is a particular type of temporomandibular joint disorder. Research findings comparing various treatment approaches are scarce and controversial. Therefore, this study aimed to compare the effectiveness of ultrasound therapy, stabilization splint, TheraBite device, and masticatory muscle exercises in reducing pain intensity and improving mandibular mobility in patients with MPS.

**Methods:**

It was a single‐blind, randomized, parallel‐group, active‐controlled trial that took place between April 2023 and October 2023 at the Department of Fixed Prosthodontics, Damascus University. Patients older than 18 years old with myofascial pain accompanied by limited jaw opening and pain lasting for at least 6 months were included. Eighty patients were randomly assigned into four groups using online randomization software: ultrasound therapy, stabilization splint, TheraBite device, and masticatory muscle exercises. Only outcome assessors were masked to treatment allocation. The exercise regimen was the exercise program for patients with TMD. The following primary outcome measures were considered at the baseline (*t*_0_), at the first (*t*_1_), second (*t*_2_), and fourth (*t*_3_) week of treatment, and at the second (*t*_4_) and fifth (*t*_5_) month of follow‐up: pain intensity using the visual analogue scale, maximum interincisal opening, right lateral movement, and left lateral movement measured in millimeters.

**Results:**

The pain level changed from severe to mild at *t*_3_ in ultrasound therapy, stabilization splint, and TheraBite device groups. In the masticatory muscle exercises group, it changed to moderate, with a significant difference between ultrasound therapy (*p* = 0.012) and stabilization splint (*p* = 0.013) groups. In addition, the mandibular mobility continued to improve at the subsequent follow‐up periods (*t*_4_ and *t*_5_).

**Conclusions:**

All therapies are equally effective after 5‐month follow‐up. However, ultrasound therapy and stabilization splints have the benefit of achieving rapid improvement.

**Trial Registration:**

ISRCTN20833186.

## 1 Introduction

Temporomandibular joint disorders (TMDs) are among the most common conditions in dental clinics. TMDs is a term that covers a wide range of symptoms and signs, which is one of the most common disorders seen in the craniofacial region. In addition, it is the second cause of facial pain following odontogenic pain (Li and Leung 2021). The pathogenesis of TMDs is still not clearly defined, as it is considered a multietiological disorder. There are several predisposing factors, including genetic, hormonal, anatomical, and causative factors such as trauma, occlusal changes, and nonfunctional habits. In addition, various exacerbating factors prolong the duration of the disorders, including stress and parafunctional habits (Chisnoiu, Picos, and Popa 2015). TMD symptoms include facial pain, limited lower jaw movement, intracapsular sounds such as clicking or crepitus, tooth sensitivity of unknown cause, tooth or restoration fractures, and chronic headaches (De Rossi et al. 2014). Approximately 25% of TMDs are symptomatic, and only a few patients request treatment (Murphy et al. 2013). Myofascial pain syndrome (MPS) is a particular type of TMD, which is presented as chronic fascial pain related to trigger points in the neck and facial muscles (Golanska et al. 2021a).

Several therapies have been proposed for MPS, including psychosocial interventions, medications, occlusal adjustment, surgical and presurgical treatments, physiotherapy, splints, passive jaw movement devices, and ultrasound therapy (Zhang et al. 2020). Various designs of splints are used to treat MPS, which are considered a familiar treatment option, such as soft bite guard, localized occlusal interference splint, anterior bite plane splint, anterior repositioning splint, and stabilization splint (SS) (Albagieh et al. 2023; Zhang et al. 2020). The passive jaw motion device has been used in degenerative joint injuries of muscular origin, such as limitation of the mouth opening and difficulty in moving the jaw. In addition, it is used in masseter muscle rehabilitation after TMJ reconstructive surgeries. TheraBite passive jaw motion device works by forcing the muscles to stretch or move to a certain degree to strengthen the masticatory muscles, increase the range of movement of the lower jaw, and relieve pain (Maloney et al. 2002; McNeely, Armijo Olivo, and Magee 2006). Ultrasound therapy plays a crucial role in cases of myofascial pain, especially if the condition is accompanied by spasms and stiffness of the masticatory muscles, as well as articular disc displacement of muscular origin and degenerative injuries of the joint. Ultrasound therapy accelerates healing by increasing blood flow in the treated area, reduces pain by reducing swelling and edema, and relieves underlying stress within the muscles, ligaments, and tendons (Xia et al. 2017; Yıldırım 2018). Therapeutic jaw exercises are widely accepted among MPS patients because they are effective in reducing headache and pain intensity. In addition, therapeutic jaw exercises are cost‐effective when compared to other treatment approaches (Armijo‐Olivo et al. 2016a; Lindfors, Magnusson, and Ernberg 2020; Simões, da Silva, and Magesty 2023). However, research findings comparing the previous treatment approaches are scarce and controversial (Armijo‐Olivo et al. 2016a; McNeely, Armijo Olivo, and Magee 2006; Xia et al. 2017). Therefore, this study aimed to compare the effectiveness of ultrasound therapy, SS, TheraBite device, and masticatory muscle exercises in reducing pain intensity and improving mandibular mobility in patients with MPS.

## 2 Patients and Methods

### 2.1 Study Design and Patient Enrollment

It was a single‐blind, randomized, parallel‐group, active‐controlled trial with four arms. This study took place between April 2023 and October 2023 at the Department of Fixed Prosthodontics, Damascus University, and it was conducted by the Declaration of Helsinki 2013 (Parsa‐Parsi 2022) and the CONSORT statement (Alkhaqani 2023). This trial was registered and approved by the ISRCTN registry (ISRCTN20833186) on October 11, 2023. Ethical approval was obtained from the Biomedical Research Ethics Committee (N1771).

The inclusion criteria were as follows:

1. Patient with myofascial pain with limited opening according to the Diagnostic Criteria for Temporomandibular Disorders (DC/TMD) (Schiffman and Ohrbach 2016).
2. Visual analogue scale (VAS) score ≥4, with pain lasting for at least 6 months (Williamson and Hoggart 2005).
3. Patients older than 18 years.

The exclusion criteria were as follows:

1. Patient with fixed or removable prosthesis.
2. Patient with systemic diseases.
3. Patient taking analgesics and/or muscle relaxants over the past 24 h.
4. Patient taking analgesics and/or muscle relaxants throughout the treatment.
5. The patient had already undergone MPS treatment.
6. Patient with polyarthritis, osteoarthritis, or arthralgia.

**Figure 1:**
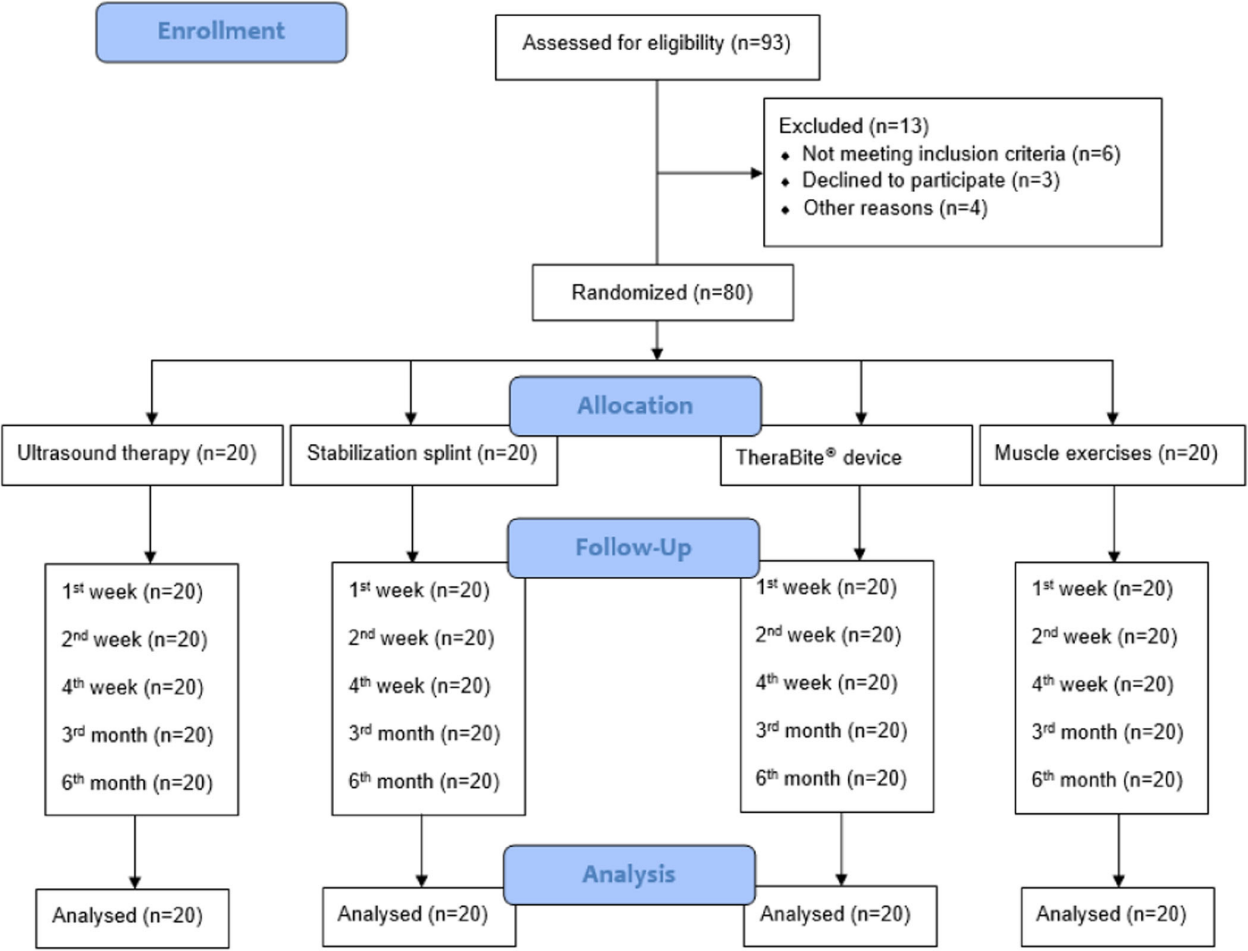
CONSORT flow diagram.

The CONSORT flow diagram is presented in Figure 1. Ninety‐three patients were assessed for eligibility, and 80 were randomly assigned into four groups according to the approach used for MPS treatment:

Group 1: Ultrasound therapy (*n* = 20).

Group 2: Control group, SS (*n* = 20).

Group 3: TheraBite device (TheraBite Jaw Motion Rehabilitation System; Atos Medical, Munich, Germany) (*n* = 20).

Group 4: Masticatory muscle exercises (*n* = 20).

### 2.2 Allocation

Randomization was performed using a simple randomization method in a ratio of 1:1:1:1, using online randomization software (https://www.randomizer.org/). The number of sets generated was four, with 20 patients per set. The number range was from 1 to 80, and each number in a set remained unique.

### 2.3 Blinding

This was a single‐blind trial where outcome assessors were masked to the treatment allocation.

### 2.4 Interventions

#### 2.4.1 Ultrasound Therapy

The patient has undergone ultrasound therapy sessions for 4 weeks at a rate of 3 weekly sessions. Each treatment session includes:

1. Warm compress for 10 min.
2. Applying ultrasound waves to the facial muscle areas with contiguous spiral movements, with a frequency of 3 MHz and an intensity of 1 W/cm^2^, for 5–10 min.
3. Muscle massage for 10 min is performed by applying circular movements with light pressure on the area around the joint and sweeping movements from the middle of the forehead toward the earlobe and from the middle of the chin toward the earlobe (Al‐Ani and Gray 2021; Morishita et al. 2014).

#### 2.4.2 SS

A full‐coverage maxillary SS was made of acrylic resin (Resilit‐S; Erkodent, Baden‐Württemberg, Germany) with a thickness of 1.5 mm. It covers approximately 1/3 of the buccal and palatal surfaces of the maxillary teeth. The patient was asked to wear the SS 8 h at night daily for 4 weeks (Alajbeg et al. 2020; Al‐Ani and Gray 2021).

#### 2.4.3 TheraBite Device

TheraBite passive motion device was used for 4 weeks in daily use. The bite pad was inserted into the mouth, and the device was opened by pushing the lever arm to the detected opening for 15 mm. The patient was instructed to bite down and hold for 10 s and rest for 30 s. Each session consisted of 10 bites (Atos Medical 2019).

#### 2.4.4 Masticatory Muscle Exercises

Each exercise is performed in the morning and evening for 1 min daily for 4 weeks. The masticatory muscle exercise program was as follows.

##### 2.4.4.1 Vertical Movement

The hand is placed under the chin, and the mouth is opened to half maximum. The movement is resisted for 10 s, followed by a rest, then repeated five times.

##### 2.4.4.2 Lateral Movement

The hand is placed on the side of the chin, opposite to the side of the injury, and the jaw is moved toward the midline. The movement is resisted for 10 s, followed by a rest, then repeated five times.

The patient is asked to stand in front of a mirror, open the mouth to the maximum comfortable range, and then close it. Appropriate pressure is applied to open the jaw straight without deviation.

The patient is asked to open the mouth slightly and to place the tongue on the buccal surface of the upper teeth, opposite to the side of the injury. The movement is resisted for 10 s, followed by a rest, then repeated five times.

##### 2.4.4.3 Protrusive Movement

The tongue depressor is placed between the teeth of the upper and lower jaws at an angle of 45°, then the lower jaw is slid over it to the maximum forward position, and the movement must occur straight. The movement is resisted for 10 s, followed by a rest, and then repeated five times (Al‐Ani and Gray 2021).

Patients were provided with a printed sheet of paper during treatment. The patients put a checkmark every time they performed the treatment as required, belonging to the different study groups.

### 2.5 Procedure

The following primary outcome measures were considered at the baseline (*t*_0_), at the first (*t*_1_), second (*t*_2_), and fourth (*t*_3_) week of treatment, and at the second (*t*_4_) and fifth (*t*_5_) month of follow‐up.

#### 2.5.1 Pain Intensity

VAS was used to evaluate pain intensity. Each patient was asked to record their current level of pain by marking a point on the VAS line that represents their pain intensity (Heller, Manuguerra, and Chow 2016).

#### 2.5.2 Maximum Interincisal Opening

Each patient was instructed to open their mouth to the maximum interincisal opening (MIO); it was measured from the incisal edge of the right maxillary central incisor to the incisal edge of the right mandibular central incisor in millimeters (Alajbeg et al. 2020; Deregibus 2021).

#### 2.5.3 Right Lateral Movement

The patient was asked to move their mandible to the right at the maximum comfortable extent, and the right lateral movement (RLM) was measured as the horizontal distance between the maxillary midline to the mandibular midline in mm (Deregibus 2021).

#### 2.5.4 Left Lateral Movement

The patient was instructed to slide their mandible to the left at the maximum comfortable extent, and the left lateral movement (LLM) was measured as the horizontal distance between the maxillary midline to the mandibular midline in mm (Deregibus 2021).

The primary outcome measures were assessed by two independent blinded clinicians. Cohen's *κ* coefficient values of intraexaminer and interexaminer reliability were >0.8.

### 2.6 Sample Size Calculation and Statistical Analysis

Sample size calculation was performed using G*Power version 3.1.9.4 (Heinrich Hein Universität Düsseldorf, Germany). A sample size of 80 patients achieved a small effect size *f* (0.37), 80% power (1 − *β* error probability), and a significance level of 0.05 (Serdar et al. 2021). Statistical analysis was performed using the IBM SPSS software version 26 (IBM Corp., Armonk, NY, USA). Data were presented as mean ± standard deviation (SD) since they were continuous variables. The Kruskal–Wallis test was run to compare the study groups, as the Kolmogorov–Smirnov test revealed that data were not normally distributed. Multiple comparisons were performed when the overall test showed significant differences across the samples.

## 3 Results

Ninety‐three patients were assessed for eligibility, and 80 were randomly assigned into four groups (Figure 1). The mean age of the patients was 30.07 years (SD 7.51; range, 20–45 years), and more than half of them were female (*n* = 48; 60%) (Table 1). The results of the Kruskal–Wallis test for comparison between the study groups at different time points in terms of pain intensity and mandibular mobility are listed in Table 2. Data were homogeneous at the baseline since no statistical significance was detected between the study groups at *t*_0_.

**Table 1:** Baseline demographic characteristics for each group.

Groups	*n*	Male		Female		Age	
		*n*	%	*n*	%	Mean	SD
Ultrasound therapy	20	6	7.5	14	17.5	31.00	9.04
Stabilization splint	20	6	7.5	14	17.5	28.20	6.16
TheraBite device	20	16	20	4	5	31.20	8.34
Masticatory muscle exercises	20	4	5	16	20	29.90	7.46
Total	80	32	40	48	60	30.07	7.51

**Table 2:** Results of the Kruskal–Wallis test for comparison between the study groups at different time points in terms of pain intensity and mandibular mobility.

Time points	Variables	Ultrasound therapy (mean ± SD)	Stabilization splint (mean ± SD)	TheraBite device (mean ± SD)	Masticatory muscle exercises (mean ± SD)	*p* value
*t*_0_	VAS	8.7 ± 0.9	8 ± 0.7	8 ± 1.1	8.4 ± 0.7	0.206
	MIO	30.9 ± 6.2	30.4 ± 4.2	32.6 ± 5.8	31.6 ± 3.7	0.799
	RLM	8.2 ± 1.9	7.3 ± 0.8	8.7 ± 2.5	8.1 ± 1.5	0.703
	LLM	8.3 ± 2.3	7.2 ± 0.9	8.5 ± 2.3	7.9 ± 2.0	0.665
*t*_1_	VAS	6.1 ± 0.9	6.3 ± 0.8	6.8 ± 1.1	7.4 ± 1.2	0.059
	MIO	32.6 ± 5.4	32.5 ± 3.7	33.7 ± 5.6	32.6 ± 3.2	0.918
	RLM	9.3 ± 1.6	8.9 ± 1.1	9.5 ± 1.6	8.4 ± 1.4	0.342
	LLM	9.8 ± 1.4	9.4 ± 1.2	9.5 ± 1.6	8.5 ± 1.4	0.223
*t*_2_	VAS	2.6 ± 1.1	2.7 ± 1.6	3.5 ± 1.4	5.5 ± 1.9	0.002*
	MIO	35.5 ± 3.7	35.2 ± 3.0	36.9 ± 4.5	35 ± 3.5	0.581
	RLM	10.1 ± 0.9	10.1 ± 0.9	10.3 ± 1.2	8.9 ± 1.4	0.082
	LLM	10.6 ± 0.5	10.4 ± 0.7	10.6 ± 1.1	9.2 ± 0.9	0.004*
*t*_3_	VAS	1.1 ± 1.3	1.1 ±± 1.0	2.5 ± 1.9	4.7 ± 1.9	0.006*
	MIO	37.8 ± 2.4	37.4 ± 1.9	38.5 ± 3.4	37.7 ± 2.8	0.737
	RLM	10.3 ± 0.5	10.5 ± 1.0	10.8 ± 0.6	9.6 ± 1.1	0.033*
	LLM	10.8 ± 0.4	10.6 ± 0.5	11.1 ± 0.6	9.4 ± 1.2	0.002*
*t*_4_	VAS	0.6 ± 1.1	1.1 ± 0.9	1.9 ± 2.0	2.9 ± 2.5	0.085
	MIO	36 ± 3.3	37.6 ± 1.0	37 ± 4.4	36.9 ± 2.3	0.694
	RLM	10.0 ± 0.8	10 ± 0.8	9.7 ± 1.1	9.8 ± 1.0	0.946
	LLM	10.1 ± 0.7	9.9 ± 0.9	10 ± 1.2	9.5 ± 1.3	0.670
*t*_5_	VAS	0.9 ± 1.4	0.9 ± 0.9	1.5 ± 2.3	2.5 ± 2.5	0.383
	MIO	36.6 ± 3.1	38 ± 2.0	37.6 ± 3.8	37.2 ± 2.8	0.760
	RLM	10.0 ± 1.1	10.4 ± 0.7	9.6 ± 1.2	9.9 ± 1.3	0.469
	LLM	10.3 ± 0.7	10.5 ± 0.5	10.1 ± 1.2	9.4 ± 1.5	0.239

Abbreviations: LLM, left lateral movement; MIO, maximum intercisal opening; RLM, right lateral movement; *t*_0_, the baseline; *t*_1_, the first week of treatment; *t*_2_, the second week; *t*_3_, the fourth week of treatment; *t*_4_, the second month of follow‐up; *t*_5_, the fifth month of follow‐up; VAS, visual analogue scale. *Significant difference at *p* < 0.05.

### 3.1 Pain Intensity

The pain level improved after 2 weeks of treatment (*t*_2_). The level of pain changed from severe to mild at the end of the treatment (*t*_3_) in ultrasound therapy, SS, and TheraBite device groups. Nevertheless, in the masticatory muscle exercises group, the level of pain changed to moderate, with a statistically significant difference with ultrasound therapy (*p* = 0.012) and SS (*p* = 0.013) groups (Table 3). In addition, in the TheraBite device group, there was a statistically significant difference between ultrasound therapy (*p* = 0.012) and SS (*p* = 0.013) groups (Table 3). However, there was an evident decrease in the level of pain at *t*_5_, with no statistically significant difference between groups, and the level of pain was mild (Table 2).

**Table 3:** Multiple comparisons between groups at different time points.

Time points	Pairwise comparison	Variables	Standard test statistics	*p* value
*t*_2_	Ultrasound therapy versus stabilization splint	VAS	−0.277	0.782
		LLM	0.490	0.642
	Ultrasound therapy versus TheraBite device	VAS	−1.413	0.158
		LLM	−0.224	0.822
	Ultrasound therapy versus masticatory muscle exercises	VAS	−3.409	0.001*
		LLM	3.000	0.003*
	Stabilization splint versus TheraBite device	VAS	−1.136	0.256
		LLM	−0.714	0.475
	Stabilization splint versus masticatory muscle exercises	VAS	−3.133	0.002*
		LLM	2.510	0.012*
	TheraBite device versus masticatory muscle exercises	VAS	−1.996	0.046*
		LLM	3.224	0.001*
*t*_3_	Ultrasound therapy versus stabilization splint	VAS	−0.020	0.984
		RLM	−0.175	0.861
		LLM	0.673	0.501
	Ultrasound therapy versus TheraBite device	VAS	−2.506	0.012*
		RLM	−1.417	0.141
		LLM	−0.901	0.368
	Ultrasound therapy versus masticatory muscle exercises	VAS	−2.506	0.012*
		RLM	1.481	0.139
		LLM	2.745	0.006*
	Stabilization splint versus TheraBite device	VAS	−2.486	0.013*
		RLM	−1.296	0.195
		LLM	−1.573	0.116
	Stabilization splint versus masticatory muscle exercises	VAS	−2.486	0.013*
		RLM	1.656	0.098
		LLM	2.072	0.038*
	TheraBite device versus masticatory muscle exercises	VAS	0.000	1.000
		RLM	2.952	0.003*
		LLM	3.646	<0.001*

Abbreviations: LLM, left lateral movement; RLM, right lateral movement; *t*_2_, the second week; *t*_3_, the third week of treatment; VAS, visual analogue scale. *Significant difference at *p* < 0.05.

### 3.2 Mandibular Mobility

Mandibular mobility improved at *t*_2_. The masticatory muscle exercises group had a significantly lower mean LLM (9.2 ± 0.9) and (9.4 ± 1.2) at *t*_2_ and *t*_3_, respectively (*p* < 0.05) (Tables 2 and 3). However, mandibular mobility continued to improve at the subsequent follow‐up periods (*t*_4_ and *t*_5_) with no statistically significant difference between groups (Table 2).

## 4 Discussion

Pain is the first symptom that prompts the patient to seek medical advice for TMDs, especially in chronic cases or those accompanied by other symptoms such as headaches or restricted mouth opening (Eweka, Ogundana, and Agbelusi 2016). MPS is a common cause of temporomandibular pain with a prevalence of 45.3% (Golanska et al. 2021b). Studies comparing various treatment approaches for MPS are not conclusive (Armijo‐Olivo et al. 2016a; McNeely, Armijo Olivo, and Magee 2006; Xia et al. 2017). Therefore, this study aimed to compare the effectiveness of ultrasound therapy, SS, TheraBite device, and masticatory muscle exercises in reducing pain intensity and improving mandibular mobility in patients with MPS.

In this study, VAS was used to evaluate pain intensity because it is simple, reliable, sensitive to small changes within individuals, reproducible, and quick to document (Escalona‐Marfil et al. 2020). The SS was selected as the control group because it is considered the gold standard treatment for MPS due to its high clinical success according to many studies (Al‐Ani and Gray 2007). The result of this study showed that ultrasound therapy and SS were superior to masticatory muscle exercises in terms of pain relief at the end of the treatment. This result could be attributed to the fact that patients would lack compliance to stick to the masticatory muscle exercises program compared to other therapies. However, a SS was combined with exercises in the study by Chen, Ning, and Lu (2022). According to Alajbeg et al. (2020), a SS has an additional advantage over a placebo splint in reducing pain intensity in patients with orofacial pain. Furthermore, Kostrzewa‐Janicka et al. (2013) suggested that a SS was effective in relieving pain in TMD patients of myogenic origin. According to Ettlin et al. (2008), a SS redistributes the joint contact area and distance between the articular fossa and condyle, which in turn reduces the joint load. According to Oliveira et al. (2019), occlusal splint has a positive effect on postural balance in TMD patients. In addition, the previous result is in agreement with the study by Ba, Zhou, and Yu (2021), which concluded that ultrasound therapy is highly effective in reducing pain in TMD patients. However, the encouraging results of ultrasound therapy could be attributed to the “novelty effect” as people tend to evoke a better response when encountered with new experiences and technologies (Shin et al. 2019). According to Deregibus (2021), a 6‐month treatment with occlusal splint is not effective in reducing pain in MPS patients. Similarly, in the current study, ultrasound therapy and SS were superior to TheraBite device in reducing pain at the end of the treatment. However, the effectiveness of the TheraBite device is similar to that of the masticatory muscle exercises. This result is consistent with the findings of De Laat, Stappaerts, and Papy (2003), which suggested that TheraBite device is equally effective in physical therapy in reducing pain in myogenic temporomandibular disorder. However, according to Xia et al. (2017), TheraBite device has the advantage of achieving rapid pain relief in patients with intracapsular or extracapsular temporomandibular disorder patients. In addition, according to Khamis and Abdel Wahab (2020), the use of a TheraBite device decreases the level of pain after 4 weeks in patients with trismus post‐maxillofacial surgeries. TheraBite device is mainly used as a rehabilitation system after facial surgery, which affects the jaw and masticatory muscles to increase mouth opening and muscle rehabilitation (Khamis and Abdel Wahab 2020). A possible explanation for those controversial results is that the treatment approaches used are highly dependent on the patient's compliance and performance. In addition, pain relief is not necessarily related to the change in the vertical dimension and condyle position, but it could be attributed to behavioral, cognitional, and sensorial factors (Alajbeg et al. 2020).

The result of the current study showed that mandibular mobility improved at the end of the treatment. This result is in agreement with the findings of Ba, Zhou, and Yu (2021) suggesting that ultrasound therapy improves jaw functions in the short and long term. Thus, the ultrasound therapy device has the advantage of achieving rapid mandibular mobility improvement in MPS patients. The previous result is in contrast with the findings of Zhang et al. (2021), which suggested that occlusal splint does not have a clear superiority over masticatory muscle exercises in terms of mandibular mobility improvement in painful TMD patients. In the current study, the TheraBite device group has a significantly higher mean RLM compared to the masticatory muscle exercises group. According to Charters et al. (2022), the TheraBite device is the most commonly used device for trismus patients and is efficient in improving maximal interincisal opening. However, the results of this study concluded that after a 5‐month follow‐up, there was an evident decrease in the level of pain in all study groups. In addition, mandibular mobility continued to improve during the subsequent follow‐up periods, suggesting that all therapies are equally effective after a 5‐month follow‐up. However, masticatory muscle exercises are a more cost‐effective and conservative alternative to the other treatment methods (Armijo‐Olivo et al. 2016b).

This study has limitations. First, treatment approaches in the current study were highly dependent on patients' compliance. Therefore, it is recommended to conduct further trials, which include exercise adherence measurement and analysis. Second, a long‐term follow‐up is required to validate the results. Lastly, pain intensity was measured using a subjective method, which leads to self‐report bias (Althubaiti 2016).

## 5 Conclusion

Based on our findings, the level of pain and mandibular mobility continued to improve during the subsequent follow‐up periods, suggesting that all therapies are equally effective after a 5‐month follow‐up for MPS patients. However, ultrasound therapy and SSs have the benefit of achieving rapid improvement.

## Author Contributions

K.S. carried out the experiment and drafted the manuscript. M.K. wrote the manuscript and performed the statistical analysis. I.H. planned the experiments and supervised the project. J.A.N. planned the experiments, supervised the project, and critically reviewed the manuscript. All authors have read and approved the manuscript.

## Consent

Informed consent was obtained from all subjects.

## Conflicts of Interest

The authors declare no conflicts of interest.

## Data Availability

The data sets generated during and/or analyzed during the current study are available from the corresponding author upon reasonable request.
